# Cytokine Levels and Neuropsychological Function among Patients with Attention-Deficit/Hyperactivity Disorder and Atopic Diseases

**DOI:** 10.3390/jpm12071155

**Published:** 2022-07-17

**Authors:** Shung-Jie Chang, Ho-Chang Kuo, Wen-Jiun Chou, Ching-Shu Tsai, Sheng-Yu Lee, Liang-Jen Wang

**Affiliations:** 1 Department of Child and Adolescent Psychiatry, Kaohsiung Chang Gung Memorial Hospital and Chang Gung University College of Medicine, Kaohsiung 83301, Taiwan; chopper_tonytony@hotmail.com (S.-J.C.); wjchou@cgmh.org.tw (W.-J.C.); jingshutsai@yahoo.com.tw (C.-S.T.); 2 Department of Pediatrics, Kaohsiung Chang Gung Memorial Hospital and Chang Gung University College of Medicine, Kaohsiung 83301, Taiwan; erickuo48@yahoo.com.tw; 3 Kawasaki Disease Center, Kaohsiung Chang Gung Memorial Hospital, Kaohsiung 83301, Taiwan; 4 Department of Psychiatry, Kaohsiung Veterans General Hospital, Kaohsiung 83301, Taiwan; shirleylee.ncku@gmail.com; 5 Department of Psychiatry, College of Medicine, Kaohsiung Medical University, Kaohsiung 83301, Taiwan

**Keywords:** ADHD, asthma, allergic rhinitis, atopic dermatitis, cytokine

## Abstract

Since atopic disease and inflammatory cytokines are both involved in attention deficit hyperactivity disorder (ADHD), in this study, we examined the relationship among cytokine levels, neuropsychological function, and behavioral manifestations in patients with ADHD and atopic diseases. Participants were categorized into individuals with ADHD and atopic disease (*n* = 41), those with ADHD without allergy (*n* = 74), individuals without ADHD but with allergy (*n* = 23), and those without ADHD or allergy (*n* = 49). We used the Swanson, Nolan, and Pelham IV Scale (SNAP-IV), Conners’ Continuous Performance Test (Conners CPT), and Conners’ Continuous Auditory Test of Attention (CATA) to assess patients’ behavioral symptoms, visual attention, and auditory attention, respectively. Participants’ IFN-γ, IL-1B, IL-6, IL-10, IL-13, IL-17, MCP-1, and TNF-α plasma levels were assessed using multiplex assays. We found that the prevalence rates of atopic diseases (asthma, allergic rhinitis, or atopic dermatitis) were similar between individuals with ADHD and those without ADHD. ADHD behavioral symptoms (SNAP-IV), CPT omission scores, and CATA detectability scores demonstrated significant differences between individuals with ADHD and those without ADHD, regardless of atopic diseases. However, plasma levels of cytokines (TNF-α, IFN-γ, and IL-17) were negatively correlated with inattention symptoms. This study demonstrates a potential relationship between cytokine levels and neuropsychological function among patients with ADHD and atopic diseases.

## 1. Introduction

Attention-deficit/hyperactivity disorder (ADHD), one of the most well-known neuropsychiatric diseases in childhood, has an international prevalence rate of about 3–5% [1]. ADHD is commonly characterized by symptoms of inattention, hyperactivity, and impulsivity. The correlation between atopic diseases and ADHD is still a matter of debate [2,3]. However, patients with certain allergic immune diseases, such as allergic rhinitis [4], asthma [5], and eczema [6], are believed to be at a higher risk of developing ADHD. A recent meta-analysis study indicated that atopic diseases may be involved in the severity of ADHD symptoms [7]. Furthermore, many of the previous studies have either researched diagnosis comorbidities or the severity of behavioral symptoms between ADHD and atopic diseases [8], but few studies have investigated neuropsychological performance in patients with comorbid ADHD and atopic diseases [9].

Cytokines released during allergic inflammation may hinder the development of the neurotransmitter system and prefrontal cortex, thus influencing ADHD development [10,11]. However, studies focused on inflammatory biomarkers in ADHD or atopic diseases have been inconsistent. For instance, children with ADHD have higher levels of IL-6 and IL-10 than controls [12,13,14], but some studies have shown that the IL-6 or TNF-α levels in the plasma of ADHD patients did not differ significantly from that of the control group [15]. Furthermore, IFN-γ, TNF-α, IL-16, and IL-13 levels are related to clinical manifestations and the scores of a neuropsychological test (Conner’s CPT) among children with ADHD [16,17,18]. The influence of certain cytokines, particularly IL-4, along with TNF-α, GM-CSF, IL-1, IL-2 and IL-6, has been recorded in nasal biopsies and/or nasal secretions of allergic rhinitis patients [19]. Such cytokines as IL-4, IL-13, and IL-5 have been shown to contribute to the induction of allergy and asthma [20]. Th2 cytokines like IL-4 and IL-13 are considered key players in atopic dermatitis [21].

Studies comprehensively investigating the relationship among ADHD, atopic diseases, and cytokine levels are rare. In this study, we propose that immunological dysregulation plays a role in altering atopic diseases. Therefore, we recruited individuals with and without ADHD and with or without atopic diseases to compare the cytokine levels, behavioral symptoms, and neuropsychological function between these groups.

## 2. Material and Methods

### 2.1. Study Participants

The Institutional Review Board (IRB) at Chang Gung Memorial Hospital in Taiwan approved the research protocol for this study. We enrolled suitable patients with ADHD from the Outpatient Clinic of Child Psychiatry at Kaohsiung Chang Gung Children’s Hospital in Taiwan. Before the participants and their parents or guardians were entered into this study, the study protocols were explained to them. Their written informed consent was also acquired upon their agreement.

Patients with ADHD were required to meet the following three criteria to be included: (a) a clinical diagnosis of ADHD made by a senior child psychiatrist through structured interviews utilizing the Diagnostic and Statistical Manual of Mental Disorders (DSM–5) [22,23]; (b) age in the range of 6 and 16 years; and (c) no earlier history of getting any medical treatment for ADHD. Meanwhile, the exclusion criteria of this study are described below: (a) patients who had a history of major neuropsychiatric diseases, including autism spectrum disorder, substance use disorders, intellectual disabilities, psychotic disorders, or mood disorders; and (b) patients having any major physical illnesses, like congenital diseases, severe head injury, or epilepsy.

The control group consisted of children without an ADHD diagnosis or a history of major neuropsychiatric diseases. The control children (age between 6 and 16 years) were recruited from the communities surrounding Kaohsiung Chang Gung Memorial Hospital or from children who had upper respiratory tract infections (URI) but whose symptoms were currently remitted. 

### 2.2. Atopic Profiles

Both patients with ADHD and control subjects were assessed by pediatricians in Chang Gung Children’s Hospital in Taiwan to confirm the diagnoses of atopic diseases (allergic rhinitis, asthma, or atopic dermatitis). Their parents or guardians provided information about children’s health, home environmental exposures, and other relevant factors using a modified Chinese version of the International Study of Asthma and Allergies in Childhood (ISAAC) questionnaires [24]. All participants were then categorized into the following four groups: with ADHD and atopic disease [ADHD+, Allergy+], with ADHD without allergy [ADHD+, Allergy−], without ADHD but with allergy [ADHD−, Allergy+], and without ADHD or allergy [ADHD−, Allergy−].

Participants’ blood tests were taken at about 8 o’clock in the morning. To check the levels of cytokines in plasma, a standard capture sandwich assay was developed with the Luminex Flowmetrix system (Luminex, Austin, TX, USA). We coupled each capture antibody to a different bead set (Upstate Biotechnology Beads, NY; Luminex, Upstate Biotechnology Inc., Lake Placid, NY, USA). Standards (recombinant cytokines) diluted in pooled blank plasma gathered from healthy grown-ups and test sera from patients or controls were analyzed using multiplex assays as previously reported, including IFN-γ, IL-1B, IL-6, IL-10, IL-13, IL-17, MCP-1, and TNF-α. Following a sequence, we incubated the beads with diluted standards or plasma and then with detection antibodies for 2 h each at room temperature, with biotin as a reporter for 1.5 h and with fluorescent dye-conjugated streptavidin–phycoerythrin for 30 min. We utilized a flow cytometer to estimate cytokine levels, which were then analyzed with Flowmetrix software, and then we mixed 50-μL samples with multiplexed antibody-conjugated beads prior to multichannel detection of the bead array.

### 2.3. Clinical Measurements

The Conners’ Continuous Performance Test (Conners CPT) [25] and Conners’ Continuous Auditory Test of Attention (CATA) [26] were used to assess patients’ visual and auditory attention, respectively. Previous evidence has indicated that the CPT3 and CATA neuropsychological tests provide objective information about ADHD cases [27]. The measures used in the analyses are Omissions, Commissions, and Detectability (d’). In a room designed to diminish variability in testing conditions, an experienced child psychologist individual led the Conners CPT and CATA with each patient. 

We utilized the Swanson, Nolan, and Pelham IV Scale (SNAP-IV), which is a 26-item questionnaire for assessing ADHD symptoms and severity [28]. The 26 items contain 18 that relate to ADHD symptoms (nine for inattention and nine for hyperactivity and impulsivity) and eight that pertain to oppositional defiant disorder symptoms. Every item is scored on a three-point Likert scale. The Chinese versions of the SNAP-IV parent form [29] have been reported to have good concurrent validity and reliability.

### 2.4. Statistical Analysis

The statistical software package SPSS, version 16.0 (SPSS Inc., Chicago, IL, USA), was used to perform data analysis. Two-tailed *p*-values < 0.05 were considered statistically significant. We presented variables as either the mean (standard deviation) or frequency. Serum cytokine levels all exhibited significant levels of positive skewness, and arithmetic log transformations were used to create approximate normal distributions. To compare the ratios of atopic diseases between ADHD patients and controls, we applied either chi-square test or Fisher’s exact test, as appropriate. Independent *t*-test was used to examine the age difference between the ADHD group and the control group.

Furthermore, one-way analysis of variance (ANOVA) was utilized to examine the age difference across the four groups of children (with ADHD and atopic disease (ADHD+, Allergy+), with ADHD without allergy (ADHD+, Allergy−), without ADHD but with allergy (ADHD−, Allergy+), and without ADHD or allergy (ADHD−, Allergy−)). We adopted multivariate analysis of covariance (MANCOVA) to compare clinical characteristics and cytokine levels among the aforementioned four groups. We set age and gender as covariates in the MANCOVA model. LSD post-hoc analyses were used for multiple comparison adjustments. Pearson’s correlation was performed to investigate relationships between cytokine levels, ADHD clinical symptoms, and neuropsychological performance (CPT3 and CATA).

## 3. Results

### 3.1. Demographic Data

For this study, we recruited 115 children with ADHD (80% male, mean age: 8.9 years old) and 72 control subjects (59.7% male, mean age: 9.9 years old). The characteristics of the ADHD and control groups are summarized in Table 1. Regarding the distribution of atopic diseases, 24.3% and 26.4% of ADHD children and controls had allergic rhinitis; 13.9% and 16.7% of ADHD children and controls had asthma; and 6.1% and 6.9% of ADHD children and controls had atopic dermatitis, respectively. In total, 35.7% of ADHD children and 31.9% of controls had at least one atopic disease. We observed no significant difference in distribution in any of the aforementioned atopic diseases between ADHD children and controls (*p* > 0.05).

All participants were categorized into the ADHD+ Allergy+ group (*n* = 41, with ADHD and atopic disease), ADHD+ Allergy− group (*n* = 74, with ADHD without allergy), ADHD− Allergy+ group (*n* = 23, without ADHD but with allergy), or ADHD− Allergy− group (*n* = 49, without ADHD or allergy). The characteristics of the four groups are listed in Supplementary Table S1. Of the four groups, the ADHD− Allergy− group had the oldest age (10.1 years) and the highest proportion of females (51%).

### 3.2. Clinical Assessments

**Figure 1:**
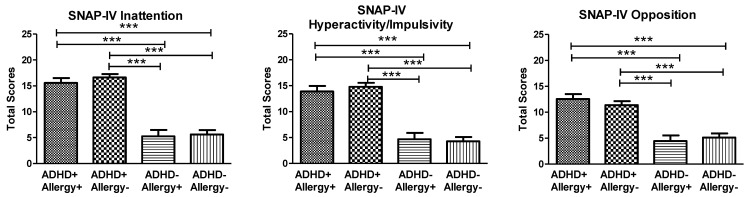
The three subscales of SNAP-IV (inattention, hyperactivity/impulsivity, and opposition scores) of children with ADHD and with atopic disease (ADHD+ Allergy+ group, *n* = 41), children with ADHD and without allergy (ADHD+ Allergy− group, *n* = 74), children without ADHD and with allergy (ADHD− Allergy+ group, *n* = 23), and children without ADHD and without allergy (ADHD− Allergy− group, *n* = 49). Multivariate analysis of covariance (MANCOVA) was used to compare clinical symptoms among the four groups of children, with age and gender as covariates. The three subscales of SNAP-IV (inattention: F_3219_ = 62.34, *p* < 0.001; hyperactivity/impulsivity: F_3219_ = 42.02, *p* < 0.001; and opposition scores: F_3219_ = 22.20, *p* < 0.001) showed significant group differences. *** *p* < 0.001 for post-hoc between group comparison.

The SNAP-IV scores of the four groups are illustrated in Figure 1. The three subscales of SNAP-IV (inattention: F_3219_ = 62.34, *p* < 0.001; hyperactivity/impulsivity: F_3219_ = 42.02, *p* < 0.001; and opposition scores: F_3219_ = 22.20, *p* < 0.001) revealed significant group differences, and we observed a similar distribution among the four groups. The post-hoc analyses showed that the children with ADHD (ADHD+ Allergy+ group and ADHD+ Allergy− group) demonstrated a higher severity of ADHD core symptoms than children without ADHD (ADHD− Allergy+ group and ADHD− Allergy− group), regardless of the presence of atopic diseases.

**Figure 2:**
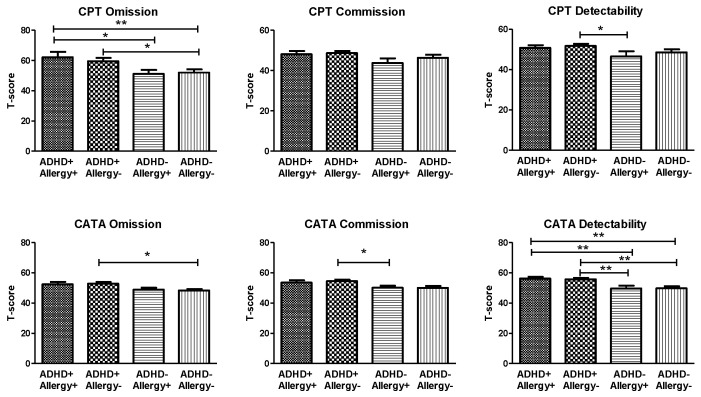
The visual attention (measured by Conner’s CPT) and auditory attention (measured by CATA) of children with ADHD and with atopic disease (ADHD+ Allergy+ group, *n* = 41), children with ADHD and without allergy (ADHD+ Allergy− group, *n* = 74), children without ADHD and with allergy (ADHD− Allergy+ group, *n* = 23), and children without ADHD and without allergy (ADHD− Allergy− group, *n* = 49). Multivariate analysis of covariance (MANCOVA) was used to compare CPT and CATA indices among the four groups of children, with age and gender as covariates. The CPT Omission scores (F_3219_ = 2.97, *p* = 0.033), CATA Omission scores (F_3219_ = 3.58, *p* = 0.015), and CATA Detectability scores (F_3219_ = 8.53, *p* < 0.001) showed significant group differences. * *p* < 0.05, ** *p* < 0.01 for post-hoc group comparison. The CATA Omission scores in the ADHD+ Allergy− group were higher than those in the ADHD− Allergy− group. Furthermore, the CATA Commission scores in the ADHD+ Allergy− group were higher than those in the ADHD− Allergy+ group. Regardless of the presence of atopic diseases, children with ADHD (ADHD+ Allergy+ group and ADHD+ Allergy− group) showed higher CATA Detectability scores than children without ADHD (ADHD− Allergy+ group and ADHD− Allergy− group).

The visual attention (measured by Conner’s CPT) and auditory attention (measured by CATA) of the four groups are shown in Figure 2. The CPT Omission scores (F_3219_ = 2.97, *p* = 0.033), CATA Omission scores (F_3219_ = 3.58, *p* = 0.015), and CATA Detectability scores (F_3219_ = 8.53, *p* < 0.001) indicated significant group differences. However, the CPT Commission scores (F_3219_ = 2.15, *p* = 0.095), CPT Detectability scores (F_3219_ = 1.91, *p* = 0.128), and CATA Commission scores (F_3219_ = 2.54, *p* = 0.057) revealed no significant differences between groups. The post-hoc analyses revealed that omission scores of the CPT in the ADHD+ Allergy+ group were higher than those in the ADHD− Allergy+ and ADHD− Allergy− groups; meanwhile, the CPT Omission scores in the ADHD+ Allergy− group were higher than those in the ADHD− Allergy− group. The CPT Detectability scores in the ADHD+ Allergy− group were higher than those in the ADHD− Allergy+ group.

### 3.3. Immunological Profile

**Figure 3:**
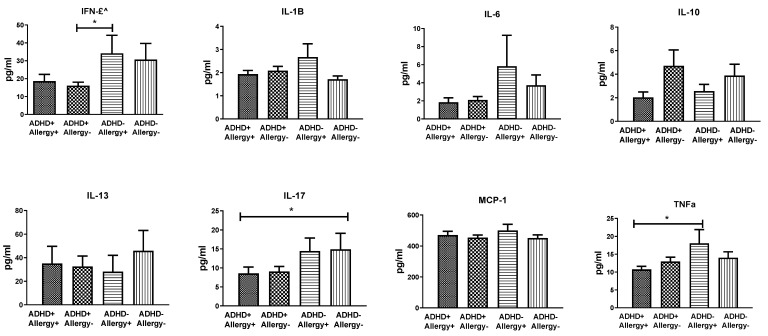
The plasma levels of IFN-γ, IL-1B, IL-6, IL-10, IL-13, IL-17, MCP-1, and TNF-α of children with ADHD and with atopic disease (ADHD+ Allergy+ group, *n* = 41), children with ADHD and without allergy (ADHD+ Allergy− group, *n* = 74), children without ADHD and with allergy (ADHD− Allergy+ group, *n* = 23), and children without ADHD and without allergy (ADHD− Allergy− group, *n* = 49). Multivariate analysis of covariance (MANCOVA) was used to compare cytokine levels among the four groups of children, with age and gender as covariates. No significant group differences were found in the plasma levels of IFN-γ (F_3219_ = 1.84, *p* = 0.142), IL-1B (F_3219_ = 1.43, *p* = 0.237), IL-6 (F_3219_ = 0.87, *p* = 0460), IL-10 (F_3219_ = 1.12, *p* = 0.344), IL-13 (F_3219_ = 0.05, *p* = 0.986), IL-17 (F_3219_ = 0.85, *p* = 0.247), MCP-1 (F_3219_ = 0.70, *p* = 0.555), and TNF-α (F_3219_ = 2.12, *p* = 0.100).* *p* < 0.05 for post-hoc group comparison.

The plasma levels of IFN-γ, IL-1B, IL-6, IL-10, IL-13, IL-17, MCP-1, and TNF-α among the four groups are displayed in Figure 3. We observed no significant group differences in the plasma levels of IFN-γ (F_3219_ = 1.84, *p* = 0.142), IL-1B (F_3219_ = 1.43, *p* = 0.237), IL-6 (F_3219_ = 0.87, *p* = 0460), IL-10 (F_3219_ = 1.12, *p* = 0.344), IL-13 (F_3219_ = 0.05, *p* = 0.986), IL-17 (F_3219_ = 0.85, *p* = 0.247), MCP-1 (F_3219_ = 0.70, *p* = 0.555), and TNF-α (F_3219_ = 2.12, *p* = 0.100). Nevertheless, the post-hoc tests revealed that IFN-γ levels in the ADHD+ Allergy− group were lower than those in the ADHD− Allergy+ group. The IL-17 levels in the ADHD+ Allergy+ group were lower than those in the ADHD− Allergy− group. In addition, the TNF-α levels in the ADHD+ Allergy+ group were lower than those in the ADHD− Allergy+ group. The plasma levels of IL-1B, IL-6, IL-10, IL-13, and MCP-1 did not differ significantly among the four groups. Age had no significant effects on the aforementioned cytokine levels, but sex had significant effects on IFN-γ, IL-1B, and IL-17.

Among all participants, the plasma levels of IL-17 were negatively correlated to SNAP-IV inattention scores (r= −0.197, *p* = 0.008) and CATA Commission scores (r= −0.183, *p* = 0.014). The levels of TNF-α (r= −0.186, *p* = 0.013) and IFN-γ (r= −0.218, *p* = 0.003) also showed negative correlation with SNAP-IV inattention scores. We found no significant correlation between levels of IL-1B, IL-6, IL-10, IL-13, and MCP-1, ADHD clinical symptoms, and neuropsychological tests.

## 4. Discussion

Our results demonstrated the prevalence rates of atopic diseases (asthma, allergic rhinitis, or atopic dermatitis) to be comparable between ADHD patients and control groups. Three dimensions of ADHD symptoms, surveyed utilizing the SNAP-IV, showed significant differences between participants with ADHD and without ADHD, regardless of the presence of atopic diseases. Compared with their counterparts, children with ADHD are more likely to have allergic conjunctivitis, allergic rhinitis, asthma, and atopic dermatitis [30,31]. A meta-analysis research demonstrated that atopic diseases were related to not only ADHD but also ADHD symptoms’ severity. The relation was even noticed in children with subthreshold ADHD, demonstrating that atopic diseases may influence the spectrum of ADHD symptom severity [7]. However, a time-series study demonstrated that the connection between ADHD and atopic diseases in children was discovered to be heterogeneous within the study population [32]. In our study, we found no differences in ADHD symptoms between ADHD patients with or without atopic diseases or among children without ADHD.

The performance of neuropsychological tests (visual attention measured by Conner’s CPT and auditory attention measured by CATA) was less consistent with ADHD behavioral symptoms. However, higher CPT Omission scores and CATA Detectability scores were observed in children with ADHD (ADHD+ Allergy+ group and ADHD+ Allergy− group), regardless of the presence of atopic diseases. A previous study compared attention deficits among patients with allergic rhinitis (AR), patients with ADHD, and healthy controls [9]. The results indicated that, when compared to the control group, allergic rhinitis patients had significant increases in perseverations in CPT and commission errors, while the ADHD group had the poorest outcome in variability and hit RT standard error [9]. Our findings suggest that visual attention measured by Conner’s CPT and auditory attention measured by CATA are associated with ADHD diagnosis, regardless of the presence of atopic diseases.

Regarding the plasma level of cytokines, IL-1B, IL-6, IL-10, IL-13, and MCP did not differ significantly between the four study groups. Meanwhile, cytokines TNF-α, IFN-γ, and IL-17 were negatively correlated with ADHD symptoms. Previous studies have also shown that TNF-αplasma levels in the ADHD group were significantly lower than those in the control group [33,34]. However, another study demonstrated that TNF-α did not differ significantly between the ADHD group and the control group, nor was there significant correlation with parent-assessed ADHD symptoms [35]. In an animal study, anti-TNF-αtreatment attenuated proteoglycan-induced arthritis progress and adjusted gut microbiota and intestinal barrier function [36]. Previous studies also reported significantly higher levels of IL-6 in ADHD patients than in controls [13,33,35]. One study revealed that increased variability of neuropsychological tests was related to lower TNF-α but to higher IFN-γ levels [17]. The role of IL-17 has been previously shown to be involved in the pathogenesis of neuroinflammatory and neurodegenerative diseases [37]. The discrepancy in the findings of our study may be associated with the small sample size and varieties in characteristics of the patients, such as environment and allergens. The dynamic roles of TNF-α, IFN-γ, and IL-17 have also been reported in the maintenance of homeostasis and neuroprotection in active neuroinflammation [38,39,40]. The correlation between cytokines, ADHD, and atopic diseases remains inconsistent among studies, and more studies are needed to further clarify the correlation [12]. Additional evidence is warranted to clarify whether the production of varied cytokines is involved in the comorbidities of ADHD and atopic diseases.

### Strengths and Limitations

This study is the first to divide patients into four group (ADHD and allergy) to investigate the possible effects on cytokines and neuropsychological tests. Another strength of this study includes adequate blood samples that measured eight biomarkers. Nevertheless, several limitations should be noted in this study. First, its sample size was small, with a notable disparity of the sample sizes across the four groups. This issue may reduce statistical power for studying behavioral measures and their correlates. Second, age and sex were not perfectly matched among the patients with or without ADHD or atopic diseases. Age and sex differences may be confounding factors of cytokines and psychological function. Third, atopic diseases were based on clinical diagnosis and the ISAAC, but IgE levels were not collected in this study. Cytokine levels other than plasma were not examined (e.g., sputum), and some essential type 2 cytokines, such as IL-4 and IL-5, were not examined in this study. Atopic diseases thus may have been misclassified. Fourth, the influence of atopic disease on cytokines or psychological function was not individually analyzed. Participants were mainly outpatients from a single hospital, and the age range was limited to ages 6 to 16 years.

## 5. Conclusions

The findings in this study indicate that the prevalence rates of atopic diseases (asthma, allergic rhinitis, or atopic dermatitis) are comparable between patients with ADHD and control groups. ADHD behavioral symptoms, CPT Omission scores, and CATA Detectability scores showed significant differences between individuals with ADHD and without ADHD, regardless of atopic diseases. However, the plasma levels of certain cytokines (IL-17, IFN-γ, and TNF-α) were inversely correlated with ADHD inattention symptoms. This study demonstrates a potential relationship between allergic immune function and ADHD, and we suggest surveying for atopic diseases and immune function among children with attention deficits.

## Figures and Tables

**Table 1:** Characteristics and allergic diseases of children with ADHD and control subjects.

Variables	ADHD (N = 115)		Controls (N = 72)		Statistics	*p*-Value
	Mean or N	SD or %	Mean or N	SD or %		
Age (years)	8.9	2.3	9.9	2.6	t =2.543	0.012
Sex					χ^2^ = 9.069	0.003
Female	23	20.0	29	40.3		
Male	92	80.0	43	59.7		
Allergic diseases						
Allergic rhinitis	28	24.3	19	26.4	χ^2^ = 0.098	0.754
Asthma	16	13.9	12	16.7	χ^2^ = 0.264	0.608
Atopic dermatitis	7	6.1	5	6.9	χ^2^ = 0.054	0.816
At least one allergic disease	41	35.7	23	31.9	χ^2^ = 0.270	0.603

Note: Data are expressed as mean ± SD or *n* (%). The *t*-value and *p*-value were calculated using the independent *t*-test; the χ^2^ and *p*-value were calculated using chi-square test.

## Data Availability

The data of the current study are available from the corresponding author on reasonable request.

## Supplementary Materials

The following supporting information can be downloaded at: https://www.mdpi.com/article/10.3390/jpm12071155/s1, Table S1. Characteristics of children with ADHD and with atopic disease (ADHD+ Allergy+ group, n = 41), children with ADHD and without allergy (ADHD+ Allergy− group, n = 74), children without ADHD and with allergy (ADHD− Allergy+ group, n = 23), and children without ADHD and without allergy (ADHD− Allergy− group, n = 49).
