# Supplementary material for: Cytokine Levels and Neuropsychological Function among Patients with Attention-Deficit/Hyperactivity Disorder and Atopic Diseases
Source: J Pers Med. 2022 Jul 17;12(7):1155. doi: 10.3390/jpm12071155 (PMC9316989; doi:10.3390/jpm12071155)
Supplement: Supplementary file 1 [file jpm-12-01155-s001.zip › jpm-1782768-supplementary.pdf]

Supplementary Table S1. Characteristics of children with ADHD and with atopic disease (ADHD+ Allergy+ group, n=41), children with ADHD and without allergy (ADHD+ Allergy- group, n=74), children without ADHD and with allergy (ADHD- Allergy+ group, n=23), and children without ADHD and without allergy (ADHD- Allergy- group, n=49).

	ADHD+ Allergy+ N=41	ADHD+ Allergy- N=74	ADHD- Allergy+ N=23	ADHD- Allergy- N=49	Statistics	p-value
Age (years), mean (SD)	9.0 (2.3)	8.9 (2.3)	9.3 (2.6)	10.1 (2.6)	$F = 2.756$	0.044*
Sex, N (%)					$\chi^2 = 18.008$	0.003*
Female	9 (22.0)	14 (18.9)	4 (17.4)	25 (51.0)		
Male	32 (78.0)	60 (81.1)	19 (82.6)	24 (49.0)		

Note: Data are expressed as mean ± SD or n (%), The F-value and p-value were calculated using one-way analysis of variance (ANOVA), The χ^2 and p-value were calculated using chi-square test. *p<0.05
